# Chrysanthemum transcription factor CmLBD1 direct lateral root formation in *Arabidopsis thaliana*

**DOI:** 10.1038/srep20009

**Published:** 2016-01-28

**Authors:** Lu Zhu, Chen Zheng, Ruixia Liu, Aiping Song, Zhaohe Zhang, Jingjing Xin, Jiafu Jiang, Sumei Chen, Fei Zhang, Weimin Fang, Fadi Chen

**Affiliations:** 1 College of Horticulture, Nanjing Agricultural University, Nanjing 210095, China

## Abstract

The plant-specific *LATERAL ORGAN BOUNDARIES DOMAIN* (*LBD*) genes are important regulators of growth and development. Here, a chrysanthemum class I LBD transcription factor gene, designated *CmLBD1*, was isolated and its function verified. *CmLBD1* was transcribed in both the root and stem, but not in the leaf. The gene responded to auxin and was shown to participate in the process of adventitious root primordium formation. Its heterologous expression in *Arabidopsis thaliana* increased the number of lateral roots formed. When provided with exogenous auxin, lateral root emergence was promoted. *CmLBD1* expression also favored callus formation from *A. thaliana* root explants in the absence of exogenously supplied phytohormones. In planta, *CmLBD1* probably acts as a positive regulator of the response to auxin fluctuations and connects auxin signaling with lateral root formation.

The plant-specific *LATERAL ORGAN BOUNDARIES DOMAIN* (*LBD*) genes play important roles in defining lateral organ boundaries and regulating many aspects of plant development, including root, leaf, inflorescence and embryo development^1^,^2^,^3^. The *Arabidopsis thaliana* genome harbours 42 *LBD* sequences, characterized by a shared, conserved ~100 residue LOB DNA binding domain^4^,^5^,^6^. Features of this domain have been used to classify the *LBD* family into the functionality-associated classes I and II. Genes belonging to class I (36 in *A. thaliana*) possess a conserved Cys block (C block) and Gly-Ala-Ser block (GAS block), while those in class II (6 in *A. thaliana*) harbor a conserved zinc finger-like domain^4^,^6^.

Auxin are a group of plant root growth regulators, indole acetic acid (IAA) is most common and natural auxin. 2.3.5-triidobenzoid acid (TIBA) is an auxin transport inhibitor, which is used to inhibit basipetal polar auxin transport in plants^7^,^8^,^9^,^10^. The rice loss-of-function mutant for the LOB domain carrying *LBD* family gene *OsARL1* is unable to form adventitious roots and thereby loses sensitivity to auxin, although it does form normal lateral roots^11^. The *A. thaliana* gene *AtLBD18* promotes lateral root emergence via its binding to the *EXPANSIN14* (*AtEXP14*) promoter. The over-expression of *AtEXP14* increases the number and density of lateral roots formed^12^. *AtLBD16* and *AtLBD18* act as regulators of lateral root emergence in response to auxin, while *AtLBD29* affects cell cycle progression and controls lateral root formation during auxin signaling^13^,^14^. AUXIN RESPONSE FACTORs, *AtARF7* and *AtARF19* modulate lateral root development via directly activating *AtLBD16* and *AtLBD29*^15^,^16^. Finally, *AtLBD16* and *AtLBD29* regulate lateral and adventitious root formation^17^. Although the development of both lateral and adventitious roots is largely governed by auxin, some of the transcription factors involved also respond to light^18^,^19^,^20^,^21^.

Chrysanthemum (*Chrysanthemum morifolium*) is a leading ornamental species, largely propagated through cuttings reliant on adventitious roots, but suffers insufficient rooting of leafy stem cuttings. The mechanisms and the signals that control the development of adventitious root were also less known. Importantly, *LBD* genes are important for root development in both dicots and monocots through the auxin signal cascades in root patterning^11^,^12^,^15^. However, little information is reference on the isolation and functional identification of LBD transcription factors in chrysanthemum. Here, the isolation of a class I chrysanthemum LOB domain gene *CmLBD1* is described. The gene is responsive to auxin and was shown to participate in the regulation of lateral root formation from calli when expressed transgenically in *A. thaliana*.

## Results

### Isolation of *CmLBD1*

**Figure 1:**
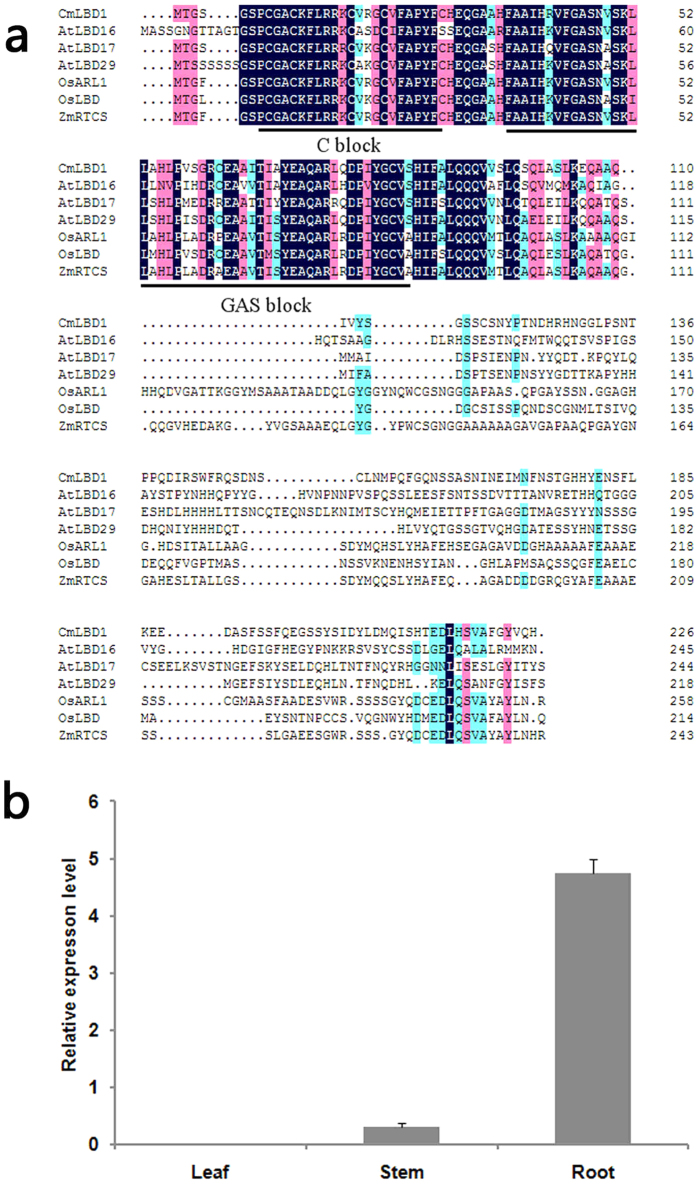
(**a**) The deduced amino acid sequence of *CmLBD1* and other genes harboring a LOB domain. The C and GAS blocks are shown underlined. The GenBank accession numbers of the genes referred to are: *AtLBD16* (NP_565973.1), *AtLBD17* (NP_850370.4), *AtLBD29* (NP_191378.1), *OsARL1* (AAV49505.1), *OsLBD* (NP_001062526.1) and *ZmRTCS* (NP_001106033.1). (**b**) The transcription of *CmLBD1* in various chrysanthemum organs. Values given as mean ± SE (*n *=* *3).

The full length *CmLBD1* cDNA comprised 842 nucleotides, with a 678 bp open reading frame encoding a 281 residue polypeptide of predicted molecular mass 57.48 kDa and pI 5.24. The polypeptide is a class I LBD member, harboring a LOB domain with both a C block and a GAS block. The former is a 22 residue sequence of the form CX_2_CX_6_CX_3_C, while the latter ends with a DPIYG motif (Fig. 1a).

### *CmLBD1* is likely involved in auxin-induced development of the adventitious root primordium

**Figure 2:**
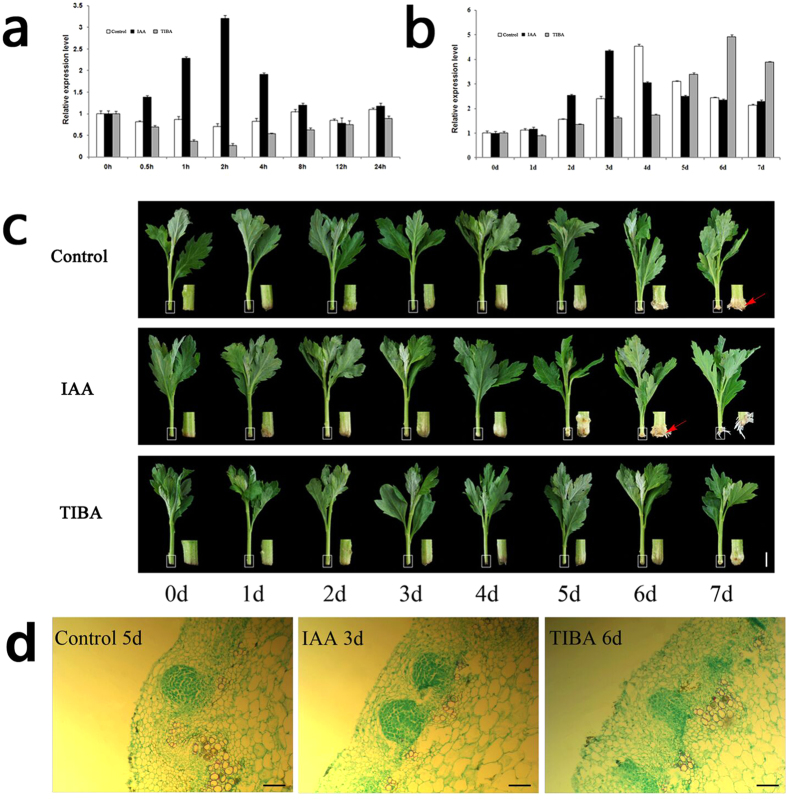
(**a,b**) Transcription profiling of *CmLBD1* in plants treated with either IAA or TIBA. Values given as mean ± SE (*n *=* *3). (b) 0–24 h, (**c**) 0–7 days. (**c**) Adventitious root primodium formation in chrysanthemum ‘Jinba’ cuttings. Primordia developed after five days in the control treatment. Exposure to IAA and TIBA respectively accelerated (four days) and delayed (seven days) this developmental stage. Bar: 1 cm. The base of the cutting is shown to the right of each image, magnified seven fold. Adventitious roots were indicated using red arrows. (**d**) Histological properties of adventitious root primodium formation in the base of cuttings. Bar: 50 μm.

*CmLBD1* transcripts were detected in the chrysanthemum stem and root, but not in the leaf. Transcript abundance was highest in the root (Fig. 1b); in the presence of auxin, the level peaked 2 h after the treatment, while in the presence of TIBA, the gene was down-regulated after 2 h, after which it slowly recovered (Fig. 2a). Once adventitious roots began to develop, the gene was markedly up-regulated, peaking after four days, and falling away once the adventitious root primordium had completely formed. In the presence of indole acetic acid (IAA), the transcription peak was brought forward by one day, while the effect of 2.3.5-triidobenzoid acid (TIBA) was to delay it by two days (Fig. 2b). Under normal conditions, early adventitious root primordium with apical meristems are visible under the microscope in five day old cuttings^22^; in the presence of auxin, this developmental stage was brought forward to three days, while in the presence of TIBA, it was delayed to six days (Fig. 2c). Histological analyses further determined the time of early adventitious root primordium in the cutting bases (Fig. 2d).

### Subcellular localization and transcriptional activation of *CmLBD1*

**Figure 3:**
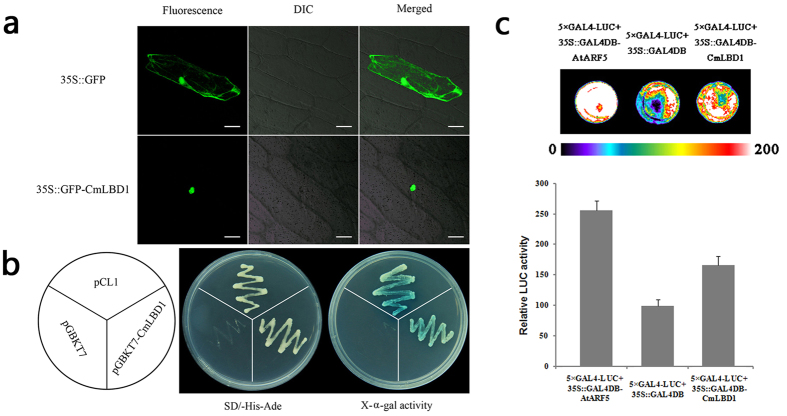
(**a**) Subcellular localization of CmLBD1 transiently expressed by the *35S::CmLBD1-GFP* transgene in onion epidermal cells. Left: dark field image, center: bright field image, right: merged image. A *35S::GFP* transgene was used as a control. Bar: 50 μm. (**b**) Transcriptional activation in yeast. pCL1 and pGBKT7 plasmids represented, respectively, the positive and negative controls. Center: SD medium lacking histidine and adenine (hemisulfate salt), right: the same medium supplemented with X-α-Gal. (**c**) Relative luciferase activities in Arabidopsis mesophyll protoplasts. 35S::GAL4DB-AtARF5, 35S::GAL4DB plasmids represented, the positive and negative controls, respectively. Above: a low-light cooled CCD imaging apparatus, below: dates of relative luciferase activities.

In onion epidermal cells transiently expressing the construct 35 S::GFP-CmLBD1, GFP accumulated most markedly in the nucleus, while the product of the control GFP transgene was deposited in both the cytoplasm and the nucleus (Fig. 3a). The yeast strain harboring the complete GAL4 domain (pCL1) was able to grow on SD/-His-Ade medium; in contrast, the negative control strain harboring the pGBKT7 vector was able to grow on Trp-deficient medium, but not on SD/-His-Ade medium. Yeasts harboring *CmLBD1* were able to activate the reporter genes *His3, Ade2* and *Mel1*, allowing the cells to grow on SD/-His-Ade medium, as shown by the pigmentation induced when the medium contained X-α-Gal (Fig. 3b). The result showed CmLBD1 had transcriptional activation activity in yeast cells *in vitro*. To further confirm the transactivation function of *CmLBD1 in vivo*, the 35 S::GAL4DB-CmLBD1 and a luciferase reporter 5 × GAL4-LUC were co-transfected into Arabidopsis protoplasts. In addition, we used 35 S::GAL4DB-AtARF5 as a positive control, and 35 S::GAL4DB as a negative control. Although the LUC activity of CmLBD1 was lower than positive control 35 S::GAL4DB-AtARF5, CmLBD1 resulted in strong LUC activity compared with negative control 35 S::GAL4DB in Arabidopsis protoplasts (Fig. 3c). These results suggested that CmLBD was an activator of transcription.

### Heterologous expression of *CmLBD1* increased lateral root growth and promoted callus growth from root explants

**Figure 4:**
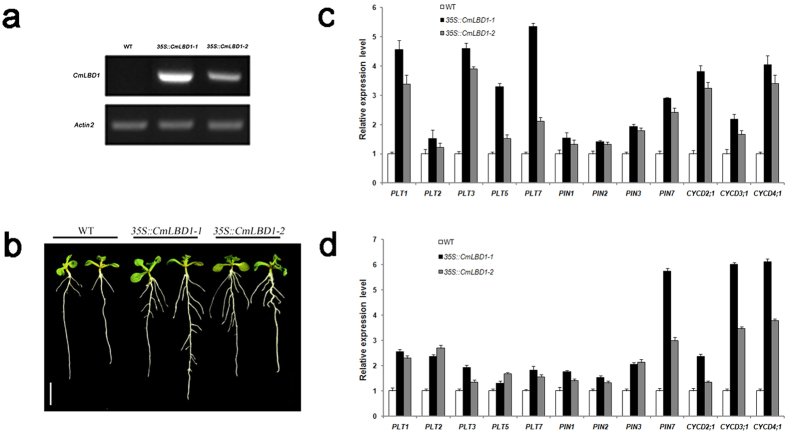
The heterologous expression of *CmLBD1* in *A. thaliana.* (**a**) *CmLBD1* expression in wild type and transgenic Arabidopsis plants in the roots of ten-day-old seedlings. Values given as mean ± SE (*n *=* *3). (**b**) The phenotype of eight day old WT and transgenic seedlings. Bar: 1 cm. qRT-PCR analysis of *PLTs, PINs* and *CYCDs* transcription in the roots of five day old (**c**) and ten day old seedlings (**d**), respectively. Values given as mean ± SE (*n *=* *3).

**Figure 5:**
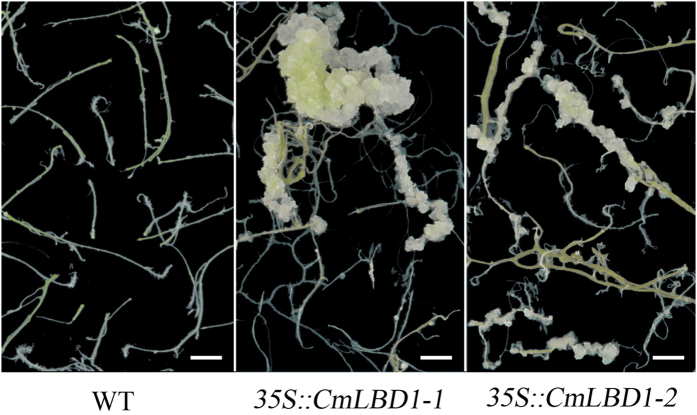
Heterologous expression of *CmLBD1* promotes callus formation from root explants. Explants of the *CmLBD1* transgenic lines and WT were cultured on B5 medium without phytohormone supplementation for 40 days. Bar: 5 mm.

Two independent transgenic *A. thaliana* lines (*35 S::CmLBD1-1* and *35 S::CmLBD1-2*) were obtained. *CmLBD1* was found in transgenic Arabidopsis *35 S::CmLBD1-1* and *35 S::CmLBD1-2*, but not in wild type Col-0 (Fig. 4a). Lateral roots were more numerous in eight day old transgenic seedlings than in wild type ones (Fig. 4b). Lateral roots were seemed swollen in the upper primary roots. But the primary root length and the average of lateral root length are almost unchanged (Table 1). A qRT-PCR analysis of the transcription in the root of a set of genes involved in lateral root initiation (*PLETHORAs, AtPLTs*; *PIN-FORMEDs, AtPINs* and D-type cyclins, *AtCYCDs*) showed they were all up-regulated in roots of 5-day-old seedlings (Fig. 4c) and 10-day-old seedlings (Fig. 4d). When root explants were cultured for ten days in the absence of exogenously supplied phytohormones, callus formation was strong in both transgenic line explants, but was suppressed in the wild type ones (Fig. 5).

### *CmLBD1* is inducible by auxin and affects lateral root formation

**Figure 6:**
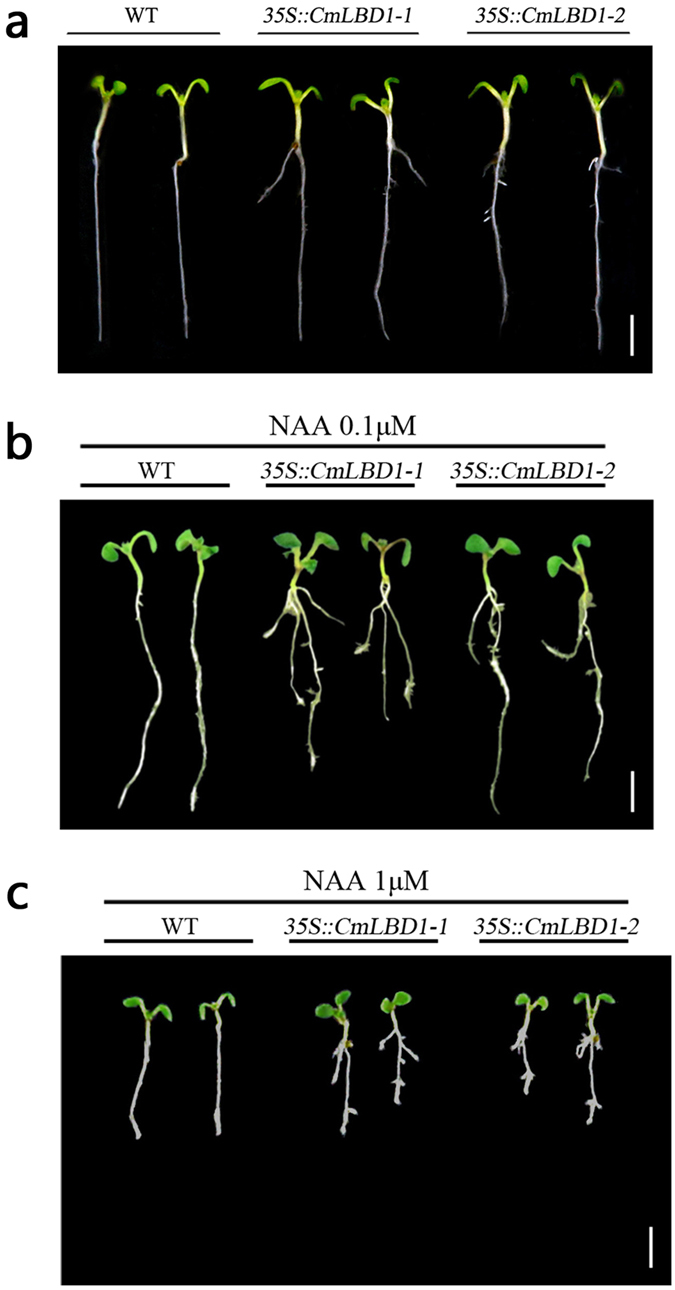
(**a**) The phenotype of six day old WT and transgenic seedlings. Bar: 1 cm. (**b,c**) Auxin-induced lateral root formation is promoted by *CmLBD1*. Three day old *CmLBD1* transgenic and WT seedlings were transferred onto a medium containing either 0.1 μM or 1 μM NAA, and scored for phenotype after 72 h. Bar: 1 cm.

The effect of providing a source of exogenous auxin 1-Naphthaleneacetic acid (NAA) on the growth of lateral roots was contrasted between wild type *A. thaliana* and the two transgenic lines. The 6-day-old seedlings of wild type and two transgenic lines which growing in 1/2 MS medium without any exogenously phytohormones as control (Fig. 6a). In the presence of either 0.1 μM or 1 μM NAA, the transgenic seedlings developed at least three lateral roots within 72 h, while the wild type seedlings produced none (Fig. 6b,c).

## Discussion

### *CmLBD1* could response to auxin signaling in Chrysanthemum

The sequence of *CmLBD1* indicates that it encodes a class I LBD transcription factor, a family of proteins which includes a number of members involved in root initiation and auxin signaling, including *AtLBD16, AtLBD17, AtLBD29, OsARL1* and *ZmRTCS*^11^,^13^ (Fig. 1a). The gene was strongly transcribed in the root and not at all in the leaf (Fig. 1b), which implies that its function is associated with root processes. In particular, the indications are that *CmLBD1* is a positive regulator of adventitious root initiation responsive to auxin signaling. In *A. thaliana*, lateral root and adventitious root initiation is responsive to auxin^23^,^24^, and the *LBD* genes *AtLBD16, AtLBD17, AtLBD18* and *AtLBD29* are all inducible when plants are grown in a medium containing the synthetic auxin 2,4-Dichlorophenoxyacetic acid (2,4-D)^25^. Similarly, tomato cotyledon explants tend to differentiate a higher number of adventitious roots when the medium contains NAA^26^. The inference is therefore that the product of *CmLBD1* contributes to the process of adventitious root primordium formation from chrysanthemum cuttings (Fig. 2c).

### *CmLBD1* localization in nucleus and acts as a transcription activator

The LBD transcription factors share a nuclear localization signal^14^,^27^,^28^. Full length, N-terminal region and C-terminal region of AtLBD16 proteins were localized predominantly in the nucleus. When basic amino acids, #113 Lys and #128 Arg changed into Thr and Ser in the coiled-coil motif, C-terminal fragment will be distributed in both the cytoplasm and the nucleus^29^. The maize rootless concerning crown and seminal roots (ZmRTCS), a LOB domin protein, localizes to the nucleus, while its paralog RTCS-LIKE (ZmRTCL) is deposited in both the nucleus and the cytoplasm^30^. The transient expression experiment showed that CmLBD1 can be localized to the nucleus (Fig. 3a), consistent with the notion that its function is to regulate the transcription of other genes. Both ZmRTCS and ZmRTCL have demonstrated a self-activation capacity in yeast^30^, and AtLOB is also capable of transcriptional activation^28^. Here, CmLBD1 was shown to be able to activate the three reporter genes *His3, Ade2* and *Mel1* in yeast, according to grow on SD/-His-Ade medium and show blue when the medium containing X-α-gal, further supporting the suggestion that *CmLBD1* is a positive regulatory factor in yeast cells (Fig. 3b). Further detection of luminescence assay showed that it might act as a transcription activator in Arabidopsis proroplasts (Fig. 3c). Being similar to *OsARL1, AtLOB* and *ZmRTCS, CmLBD1* can activate transcription in yeast and Arabidopsis proroplasts.

### Lateral roots numbers was increased with or without auxin in two transgenic *A. thaliana* lines

Although adventitious roots are morphologically similar to lateral ones, the mechanistic basis of their initiation is less well understood^31^. The heterologous expression of *CmLBD1* in *A. thaliana* resulted in a distinct phenotype, in which lateral root formation was promoted (Fig. 4b). Lateral roots were seemed swollen in upper primary root near hypocotyls. It might be a precursor of callus and form callus easier. The over-expression of *AtEXP17* (a gene regulated by *AtLBD18*) enhances lateral root formation, a result of stimulation by auxin^3^. The polar nuclear migration is inhibited in transgenic plants expressing the fusion transgene *AtLBD16-SRDX* (SRDX is a 12 residue motif which converts transcription factors to dominant repressors). Only a small number of lateral roots are formed by *Atlbd16* single and *Atlbd16/lbd18/lbd33* triple mutants, while none are formed by the *AtLBD16*-SRDX transgenic^32^. *AtARF17* negatively regulates adventitious root formation^20^,^21^. The over-expression of *AtABCB19* generates a number of adventitious roots through its enhancement of auxin transport and accumulation^33^. Here, lateral root development was promoted in the *CmLBD1* transgenic plants when they were provided with an exogenous source of auxin (Fig. 6), suggesting that *CmLBD1* controls lateral root development in response to auxin. *PLTs* are key effectors of auxin synthesis^34^,^35^,^36^ and calls formation^37^. *PINs* transport auxin from the center of the root (stele) to the new root tip, and then away again through the epidermis, which forms the basis of lateral root formation^38^,^39^,^40^. *CYCDs* controlled the G1-to-S phase of cell cycle transition and mediated pericycle responses to auxin signaling^41^,^42^,^43^. *CmLBD1* also up-regulated the transcription of *AtPLTs, AtPINs* and *AtCYCDs* genes in 5-day-old seedlings and 10-day-old seedlings (Fig. 4c,d), which were implicated in controlling lateral root formation and callus formation^35^,^44^,^45^. With heterologous expression in *A. thaliana*, lateral roots could continue to grow within in a certain time. The results indicated that *CmLBD1* might act as an important element to maintain the proliferative activity of pericycle cell.

### *CmLBD1* expression favored callus formation

Plant regeneration from *in vitro* grown callus is regulated by auxin and cytokinin. In *A. thaliana*, the genes *AtLBD16, AtLBD17, AtLBD18* and *AtLBD29* provide the necessary link between auxin signaling and regeneration^26^, while Micro160 (*MiR160*) and *AtARF10* have been identified as important regulators of shoot regeneration from *in vitro* cultures^46^. Shoots were not found from callus in the absence of exogenously supplied phytohormones medium in our experiment. *ARF10* is targeted by *MiR160*, but mechanisms are different in different tissues. The ability of *CmLBD1* transgenic lines to regenerate callus was not dependent on the provision of any phytohormones, suggesting that the expression of the transgene drives callus formation *in vitro* (Fig. 5).

Taken together, *CmLBD1*, a class I LBD transcription factor gene, played an important role in the process of adventitious root primordium formation of chrysanthemum. In *A. thaliana, CmLBD1* positive regulated lateral root formation through response auxin signaling. This strongly suggests that *CmLBD1* acts as a positive regulator and participates in root formation.

## Materials and Methods

### Plant materials and cultivation

Five to six leaf cuttings were taken from the Chrysanthemum cultivar ‘Jinba’, which is maintained by the Chrysanthemum Germplasm Resource Preserving Centre (Nanjing Agricultural University, China). The cuttings were rooted in a 1:1 mixture of perlite and vermiculite. After two weeks under the 16 h photoperiod (80-100 μmol/m^2^/s illumination) at 22 ± 1 °C conditions, the roots, stems and leaves were explored and analysed the tissue-specific transcription profiles of *CmLBD1* gene. The transcription of *CmLBD1* was monitored in cuttings held for 1 h in a liquid medium containing either 150 μM auxin IAA or 150 μM of the auxin inhibitor TIBA^47^,^48^, with a set of control cuttings placed in sterile water. Each cutting base with 8 mm in length was sampled before the transfer, then after 0.5, 1, 2, 4, 8, 12 and 24 hours, and subsequently once daily over the next six days. Samples were taken in triplicate.

### Isolation and sequencing of *CmLBD1* cDNA

RNA was isolated from leaves, stems and roots of ‘Jinba’ plants using the RNAiso reagent (TaKaRa, Tokyo, Japan) according the manufacturer’s protocol, and a 1 μg aliquot was converted into ss cDNA using M-MLV reverse transcriptase (TaKaRa). Internal fragment was identified based on other LBD genes sequences in GenBank. The full length *CmLBD1* cDNA was obtained by applying 5′-RACE and 3′-RACE PCR^49^. For the 3′ reaction, the adaptor primer dT-AP was used for the reverse transcription step and the gene-specific primers GSP3′-1/-2/-3 for the amplification step. For the 5′ reaction, the gene-specific primers GSP3′-1/-2/-3 and 5′ RACE System kit (Invitrogen, Carlsbad, CA, USA). The resulting PCR product was purified and inserted into the plasmid pMD19-T (TaKaRa) for sequencing. Primer sequences are provided in Table S1.

### Subcellular localization of *CmLBD1*

The *CmLBD1* coding sequence was amplified using a forward primer (LBD1-ENTR1A-F) and a reverse primer (LBD1-ENTR1A-R) (table S1 in file S1). The purified PCR product was restricted by *Sal* I and *Not* I and the resulting fragment inserted into the pENTR1A vector (Invitrogen) and thence into pMDC43^50^ using LR Clonase^TM^ II enzyme mix (Invitrogen). An empty vector (containing the N terminus of GFP) was used as a negative control. The recombinant plasmids were transiently expressed in onion epidermal cells, following He-driven particle bombardment (PDS-1000; Bio-Rad, Hercules, CA, USA) and a 16 h culture on Murashige-Skoog (MS) medium in the dark at 23 °C^51^. The expression of GFP was monitored by confocal laser scanning microscopy.

### Transcriptional activity analysis of *CmLBD1*

The *CmLBD1* open reading frame was amplified using the primer pair LBD1-BD-F / LBD1-BD-R, which harbor, respectively, an *Nde* I and a *Bam*H I recognition site (primer sequences given in Table S1) and then inserted into the yeast expression vector pGBKT7 (Clontech, Mountain View, CA, USA). The vector pCL1 (containing a full length copy of GAL4) was used as a positive control, and an empty pGBKT7 as the negative control. The constructs were introduced into yeast (*Saccharomyces cereviseae*) strain Y2HGold (Clontech) following the Yeastmaker Yeast Transformation System 2 protocol. The pCL1 transformants were incubated on a SD medium SD lacking leucine, while the pGBKT7 and pGBKT7-*CmLBD1* ones were incubated on a SD medium lacking tryptophan. After culturing at 30 °C for 3 d, the transgenic cell lines were transferred onto a SD medium lacking both histidine and adenine (hemisulfate salt) either in the presence or absence of X-α-Gal^52^.

Then the luminescence assay of *CmLBD1* was further examined for transactivation activity in Arabidopsis mesophyll protoplasts. The plasmid pENTR1A-*CmLBD1* previously was subjected to vector 35S::GAL4DB using LR Clonase^TM^ II enzyme mix. Arabidopsis mesophyll protoplast isolation and transformation were based on the protocol as described by Wu *et al*.^53^. 7.5 μg 35S::GAL4DB-AtARF5, 35S::GAL4DB and 35S::GAL4DB-CmLBD1 were transfected, respectively. Additional 7.5 μg GAL4-LUC as luciferase reporter plasmid were added^54^. Luciferase assay was as described by Fujikawa and Kato^55^, except D-Luciferin (for firefly luciferase; Gold Bio Technology) replaced the ViviRen Live Cell Substrate. The protoplasts were incubated in 6-well plates for 16 h in light at 23 °C. LUC images were captured by a low-light cooled CCD imaging apparatus (DU934P Andor, UK) in 96-well plate. LUC activity was measured with 10 sec integration periods (Promega, Madison, Wisconsin, USA). Counts of luminescence were quantified with a 20/20n Luminometer (Turner BioSystems). Three independent experiments were performed for each assay.

### *A. thaliana* transformation

The *CmLBD1* sequence harbored by pENTR1A was transferred into pEarleyGate103^56^ using LR Clonase^TM^ II enzyme mix, then transformed into *A. thaliana* Col-0 using an *Agrobacterium tumefaciens EHA105*-mediated floral dip method^34^. The transgene was driven by the CaMV 35S promoter. Transgenic progeny were selected by including basta herbicide in the culture medium.

### Quantitative real-time PCR (qRT-PCR) analysis

RNA was extracted from plant tissue with the RNAiso reagent (TaKaRa), and treated with DNase to remove any genomic DNA contamination. The cDNA first strand was synthesized M-MLV reverse transcriptase. Each 20 μL qRT-PCR contained 10 μL SYBR Premix Ex Taq™ II (Takara), 5 μL cDNA template (1 ng/μl) and 0.4 μL of each primer (10 μM). The handling of each PCR followed Zhu *et al*.^49^. All reactions were performed in three technical replicates and the chrysanthemum *EF1α* sequence (KF305681) was used as the reference^57^. Relative transcript abundances were calculated using the 2^−ΔΔCt^ method. Transcription profiling in the root of ten day old seedlings was also carried out for the genes *PLT1* (AT3G20840), *PLT2* (AT1G51190), *PLT3* (AT5G10510), *PLT5* (AT5G57390), *PLT7* (AT5G65510), *PIN1* (AT1G73590), *PIN2* (AT5G57090), *PIN3* (AT1G70940), *PIN7* (AT1G23080), *CYCD2;1* (AT2G22490), *CYCD3;1* (AT4G34160) and *CYCD4;1* (AT5G65420), using primer sequences identified by Feng *et al*.^44^ and Chen M-K *et al*.^58^ (see Table S1); the sequence of *AtActin2* (At3g18780) was used as the reference.

### Auxin-induced lateral root formation in transgenic plants

*A. thaliana* seedlings were grown on vertically oriented plates containing half strength MS medium, 3% w/v sucrose and 0.6% w/v agar under a 16 h photoperiod at 22 ± 1 °C. After three days, wild type and two independent transgenic *A. thaliana* seedlings were transferred to a half strength MS medium containing either 0.1 μM or 1 μM naphthalene acetic acid (NAA). The growth of lateral roots was observed after 72 h^32^.

### Callus formation *in vitro* grown root explants

Root material was sampled from the maturation zone of both wild type and *CmLBD1* transgenic seedlings cultured on half strength MS medium. The explants were transferred to B5 medium lacking phytohormones and cultured for 40 days to induce callus formation^26^.

## Additional Information

**How to cite this article**: Zhu, L. *et al*. Chrysanthemum transcription factor CmLBD1 direct lateral root formation in *Arabidopsis thaliana. Sci. Rep.* **6**, 20009; doi: 10.1038/srep20009 (2016).

## Supplementary Material

Supplementary Information

## Figures and Tables

**Table 1:** Analysis of root formation in wild type and heterologous expression of *CmLBD1* on *A. thaliana*.

	**Wild type**	**35 S::CmLBD1-1**	**35 S::CmLBD1-2**
Primary root length(cm)	18.82 ± 0.15	20.81 ± 0.14	20.33 ± 0.19
Lateral roots densities	2.45 ± 0.07	9.71 ± 0.25**	9.03 ± 0.40**
