# Supplementary material for: Chrysanthemum transcription factor CmLBD1 direct lateral root formation in Arabidopsis thaliana
Source: Sci Rep. 2016 Jan 28;6:20009. doi: 10.1038/srep20009 (PMC4730235; doi:10.1038/srep20009)
Supplement: Supplementary Information [file srep20009-s1.pdf]

Title: Chrysanthemum transcription factor CmLBD1 direct lateral root formation in *Arabidopsis thaliana*

Authors: Lu Zhu, Chen Zheng, Ruixia Liu, Aiping Song, Zhaohe Zhang, Jingjing Xin, Jiafu Jiang, Sumei Chen, Fei Zhang, Weimin Fang, Fadi Chen*

Supplementary Information

Supplementary Table S1.Primer sequences used in this study

Primer name	Sequences (5'-3')
**F**	CGCCCCCTACTTCTGCcaygarcarcargg
**R**	CCTGCTGCTGCAGGgcraadatrtg
**Oligo(dT)**	GACTCGAGTCGACATCGATTTTTTTTTTTTTTTTT
**dT-AP**	GACTCGAGTCGACATCGA
**GSP3'-1**	AGGTTCCCCTTGTGGTGCTT
**GSP3'-2**	TGAACAAGGTGCTGCCCACT
**GSP3'-3**	TAAACTCCTTGCTCACCTCCCTG
**AAP**	GGCCACGCGTCGACTAGTACGGGIIGGGIIGGGII
**AUAP**	GGCCACGCGTCGACTAGTAC
**GSP5'-1**	TGGGCAGCACCTTGTTCA
**GSP5'-2**	GGAGGTGAGCAAGGAGTT
**GSP5'-3**	TGGGATCTTGAAGTCTCG
**Full-F**	TAGTCTAAAACCTTAAACTAATC
**Full-R**	CCCAAACTGGTATTGATTCTCCC
**LBD1-RT-F**	ATGAACTTCAACTCGACGGGACA
**LBD1-RT-R**	ATCCCAAACTGGTATTGATTCTCC
**CmEF1a-F**	CCATTCAAGCGACAGACTCA
**CmEF1a-R**	TTTTGGTATCTGGTCCTGGAG
**LBD1-pENTR1A-F**	GTCGACATGACAGGATCAGGTTCC
**LBD1-pENTR1A-R**	GCGGCCGCGAATGTTGAACATAGCCGAA
**LBD1-BD-F**	CATATGATGACAGGATCAGGTTCC
**LBD1-BD-R**	GGATCCGAATGTTGAACATAGCCGAA
